# Diabetes, glucose control, glucose lowering medications, and cancer risk: A 10-year population-based historical cohort

**DOI:** 10.1186/1471-2407-12-364

**Published:** 2012-08-23

**Authors:** Rachel Dankner, Ran Balicer, Paolo Boffetta, Lital Keinan Boker, Sylvan Wallenstein, Laurence Freedman, Margalit Goldfracht, Jesse Roth, Ronald Tamler, Derek LeRoith

**Affiliations:** 1 Unit for Cardiovascular Epidemiology, The Gertner Institute for Epidemiology and Health Policy Research, Sheba Medical Center, Tel Hashomer, 52621, Israel; 2 Sackler Faculty of Medicine, School of Public Health, Department of Epidemiology and Preventive Medicine, Tel Aviv University, Ramat Aviv, Tel Aviv, 69978, Israel; 3 Patient Oriented Research, The Feinstein Institute for Medical Research Manhasset, North Shore, NY, 11030, USA; 4 Clalit Health Services Research Institute, Arlozerov St. 105, Tel Aviv, 62908, Israel; 5 School of Public Health, Ben-Gurion University of the Negev, Beer Sheba, Israel; 6 Institute for Transitional Epidemiology, Mount Sinai School of Medicine, New York, NY, USA; 7 Israel Center for Disease Control, Sheba Medical Center, Ramat Gan, Israel; 8 School of Public Health, Haifa University, Haifa, Israel; 9 Division of Endocrinology, Diabetes, and Bone Disease, Mount Sinai School of Medicine, New York, NY, USA; 10 Biostatistics Unit, Gertner Institute for Epidemiology and Health Policy Research, Sheba Medical Center, Tel Hashomer, 52161, Israel; 11 Albert Einstein College of Medicine, Yeshiva University, Bronx, NY, USA

**Keywords:** Historical prospective, Incidence, Population based, Time dependent analysis

## Abstract

**Background:**

Both diabetes and glucose-lowering medications have been associated with an increased risk of cancer incidence. This study will compare cancer incidence rates in individuals with and without diabetes; and will investigate, in individuals with diabetes, an association between glucose control and cancer incidence; and between the use of specific glucose-lowering medications, as well as no drug exposure, and cancer incidence.

**Methods/design:**

This is a population based historical cohort study of all individuals aged 21 years or older (about 2,300,000) who were insured by Clalit Health Services, the largest health maintenance organization in Israel during a ten-year study period. Four study groups will be established according to the status of diabetes and cancer at study entry, Jan 1, 2002: cancer free, diabetes free; cancer free, diabetes prevalent; cancer prevalent, diabetes free; and cancer prevalent, diabetes prevalent. Individuals without diabetes at study entry will be followed for diabetes incidence, and all four groups will be followed for specific cancer incidence, including second primary neoplasms. Glucose control will be assessed by HbA1c and by fasting plasma glucose levels. Time dependent regression models for cancer incidence will account for glucose-lowering medications as they are added and changed over the follow-up period. A large number of demographic and clinical variables will be considered, including: age, gender, BMI, smoking status, concomitant medications, glucose control (assessed by HbA1c and by fasting plasma glucose) and cancer screening tests.

**Discussion:**

Strengths of this study include the large population; high quality comprehensive data; comparison to individuals without diabetes, and to those with diabetes but not treated with glucose-lowering medications; and the extensive range of variables available for analysis. The great increases in diabetes prevalence and in treatment options render this study particularly relevant and timely. The Israeli national healthcare system, characterized by high standard and uniform healthcare, offers an advantageous environment for its conduct.

## Background

This study responds to a number of recent trends: the increasing prevalence of diabetes, the accumulating evidence of the relationship between diabetes and cancer [1] and the increasing number of options available for treating diabetes. The overall aim is to elucidate the relationship between diabetes and cancer incidence (overall and specific cancers, including second primary neoplasms). Specifically, this is a ten year nationwide population-based investigation of the associations of cancer incidence with diabetes, glucose control and glucose-lowering medications.

To the best of our knowledge, a relationship between glucose control and cancer has not yet been examined. Since glucose control has been found to associate with both micro- and macrovascular complications of diabetes, and cancer is now considered a complication of diabetes, investigation of the effect of glucose control on cancer risk is relevant. Further, data on glucose control is important for the investigation of glucose-lowering medications, since initiation of treatment and switching between treatments may be related to patients’ glucose control.

The use of glucose-lowering medications has been associated with cancer incidence. Sulfonylureas and injected insulin have been found to associate positively with cancer risk [2-5]; and metformin, which improves insulin sensitivity and lowers insulin levels, to be associated negatively with cancer risk, and to possibly have a protective role [2-7]. A number of studies reported elevated incidence of malignancy following the use of insulin glargine [8-10], though their methodologies have been seriously challenged [11-14]. In particular, the Hemkens et al. study [9] was widely criticized [15-17] for not considering such factors as current health status, type of diabetes, intensity of glucose lowering treatment, concomitant medications, BMI, glucose control (HbA1c), hormone replacement therapy, smoking status, and cancer screening. More recently, others have tried to overcome biases by comparing glargine to human insulin, while excluding other insulin analogs [18], or by including only individuals with type 2 diabetes [19]. Nevertheless, those studies were criticized for short follow-up periods. Siussa et al. [20] showed how long-term follow-up, of up to 8 years, helped distinguish between possible mechanisms for the association between glargine use and an increased risk of cancer. The proposed study, with 10 year follow-up, will assess oral glucose lowering medications, as well as insulin. We expect to reveal differential effects of glucose-lowering medications on site-specific cancer in one large population cohort.

## Methods/design

This is a large scale historical cohort study of all adult individuals insured by Clalit Health Services, the largest health maintenance organization in Israel, insuring and providing healthcare to 55% of the Israeli population (about 3.9 million people). Study entry is defined as January 1, 2002. Inclusion criteria will be: age 21 years or older at study entry and continuous insurance in Clalit Health Services from at least one year prior to study entry, January 1 2001, until the end of the study period, December 31, 2011, or until death, for those who do not survive until the end of follow-up. Of the population of over 2,300,000, over 110,000 had diabetes at study entry; and an additional 350,000 were diagnosed with diabetes during the study period (January 2002 to December 2011).

This study has three Specific Aims: First, we will compare rates of cancer incidence (overall and specific cancers, including second primary neoplasms) in individuals with and without diabetes (separately for prevalent and incident cases of diabetes). Second, we will investigate, in individuals with diabetes, the associations between measures of glucose control, as assessed by HbA1c and fasting plasma glucose, and cancer incidence (overall and specific cancers, including second primary neoplasms). Third, we will compare the incidence of cancer (overall and specific cancers, including second primary neoplasms), among individuals with diabetes who used specific glucose-lowering medications, as well as among patients with no drug exposure (periods of time during which individuals classified with diabetes did not yet purchase glucose-lowering medications, including periods on diet and exercise regimen only).

**Figure 1:**
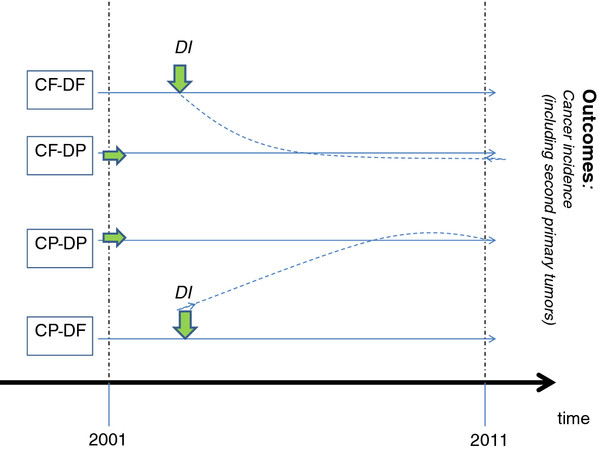
**The ten year follow-up of the four study groups, according to disease status at study entry: diabetes free (DF) or prevalent (DP), and cancer free (CF) or prevalent (CP).** For the groups that are DF at study entry, diabetes incidence (DI) will be followed and associated with outcomes (green arrows pointing down). For the groups that are DP at study entry, glucose control and diabetes medications will be followed and associated with the outcomes (green arrows pointing to the right). All four groups will be followed for the study outcome: overall and specific cancers, including second primary neoplasms.

**Figure 2:**
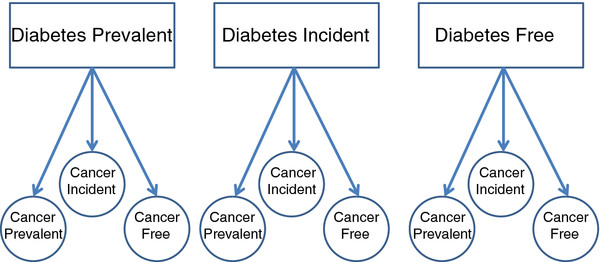
**The study cohort according to diabetes and cancer status during the ten year follow-up period.** DP - prevalent diabetes; DI - incident diabetes; DF - diabetes free; CP: prevalent cancer; CI - incident cancer; CF - cancer free.

Four study groups will be established according to the status of diabetes and cancer at study entry, Jan 1, 2002 (Figure 1): cancer free, diabetes free (CF-DF); cancer free, diabetes prevalent (CF-DP); cancer prevalent, diabetes free (CP-DF); and cancer prevalent, diabetes prevalent (CP-DP). Individuals free of diabetes at study entry will be followed for diabetes incidence, and all four groups will be followed for cancer incidence (second primaries for those with prevalent cancer). Nine groups, according to diabetes and cancer prevalence at study entry, and diabetes and cancer and incidence during the study period, will be analyzed (Figure 2).

### Diabetes classification

Prevalent diabetes was determined according to physician reports to the chronic disease registry of Clalit Health Services. Incident diabetes will be determined by any one of the following criteria: two fasting glucose readings of 126 mg/dl or above during the course of a year, HbA1c greater than 7.0%, 3 recorded purchases of glucose-lowering medications during the course of a year, diagnosis of diabetes according to the Chronic Disease Registry of Clalit Health Services, or a written diagnosis by a physician (in the community or hospital). The relatively low number of individuals classified with diabetes at study entry (prevalent diabetes) is apparently due to the more limited definition of diabetes used by Clalit Health Services prior to 2002.

### Exposure to glucose-lowering medications

In addition to the exposure to diabetes per se, individuals with diabetes will be studied for their exposure to glucose-lowering medications. A drug exposure will be defined as a minimum of 3 purchases of glucose-lowering medications during one year. Each drug exposure will define one type of treatment or a combination of treatments. An individual may be analyzed according to more than one exposure during follow-up time. Drug exposures to be investigated are: each insulin type, meglitinide derivatives, sulfonylureas, biguanides, alpha glucosidase inhibitors, thiazolidinediones, and incretins. An additional group (“no glucose-lowering medication”) will comprise periods of time during which individuals classified with diabetes did not yet purchase glucose-lowering medications.

For each glucose-lowering medication, cumulative exposure will be assessed as the total number of monthly dosages purchased. Daily dosages and daily dosages per body weight will be estimated from records of purchases. Time dependent regression models for the outcomes investigated will account for glucose-lowering medications as they are added and changed over the follow up period, adjusting for constant and time-dependent covariates.

### Potential confounders

The following variables will be included together with the time they were recorded:

1. Age, gender, BMI, type of medical insurance (basic, or basic and supplementary).

2. Smoking status (non-smoker, former smoker, current smoker)

3. Concomitant medications (types, exposure rates, and cumulative exposures), including antihypertensive, lipid lowering, beta-blockers, angiotensin converting enzyme (ACE) inhibitors

4. Comorbidities defined according to the Clalit Registry of Chronic Diseases, with focus on those associated with cancer risk (e.g. IBD, COPD, gallstones)

5. Blood pressure (mean annual values)

6. Reproductive factors: hormone replacement therapy, fertility treatments

7. Biochemical characteristics (first and last tests during follow-up, mean annual values, and number of blood tests performed annually): serum lipids (total cholesterol, triglycerides, HDL-cholesterol, VLDL-cholesterol), liver enzymes, creatinine, and urine protein

8. Clinical examinations (average annual number of visits to the treating physician during follow-up)

9. Cancer screening tests: mammography, fecal occult blood, colonoscopy, PAP smear, colposcopy, prostate screening (PSA, ultrasound)

10. Medical procedures associated with cancer risk (e.g. cholecystectomy).

We note that data on some key potential confounders (like smoking and fertility treatments) are available for only a subset of the cohort. We will conduct sensitivity analyses in these subsets to assess whether these factors indeed confound the associations between diabetes, glucose-lowering medications, and cancer.

### Outcomes

Incidence of cancer (both first and second primary neoplasms) will be the primary outcome. For diagnoses of cancer, the following data from the Israel National Cancer Registry will be linked to the study file: date of diagnosis; place of diagnosis; hospital or other reporting source; disease type, site, morphology, and stage at diagnosis; tumor behavior (in-situ, benign, malignant, borderline, and uncertain); basis of diagnosis (pathology report, clinical only, imaging devices, or based on death certificates only); and mortality data. Mortality data is continuously updated by reports from the Ministry for Internal Affairs.

### Data management

A database will be constructed for the proposed study from the Clalit Health Services data warehouse. Available are demographic data and vital status from the Israeli Ministry of Interior Affairs and the National Insurance Institute (Social Security); drug dispensing data from pharmacies; and medical data such as laboratory tests, imaging results, diagnoses and clinical data (blood pressure, weight, height, etc.) from Clalit Health Services in-house facilities, as well as from outside suppliers. The data are accessible at the member level, and linked to all registries, including the Cancer Registry, by means of a national identification number, a unique identifier possessed by all Israeli citizens. Data will be organized according to the BI (business intelligence) schema or infrastructure and are extracted using BI programs. Extraction is executed via the BO (business object) and SQL server management studio programs, depending on the complexity and diversity of the data requested from the data warehouse. BI analyst specialists will conduct the extraction and merge the data.

### Power calculation

The most conservative estimates yield at least 175,000 incident cases of cancer in the total cohort. Thus, adequate power is expected for a large number of cancer types and treatment regimens. The sample size for analysis will decrease as the number of treatment switches increases and as certain combinations become rare. Nevertheless, power will be adequate to find relatively small differences based even on subsets of 500–1000 events for a particular treatment or treatment combination. Specifically, we will have over 80% power (based on two-tailed testing at the 0.05 level) to detect a hazard ratio of 1.4 for two subsets of 6000 persons each.

### Statistical analyses

For our first specific aim, we will compare, after adjustment, all and site-specific cancer rates between individuals with and without diabetes. For the second aim, we will investigate whether metabolic control, as indicated by HbA1c and blood glucose levels, is related to cancer risk. Third, we will evaluate differences in outcomes that associate with the use of one or a combination of glucose-lowering treatments. In all of these analyses we will stratify persons with diabetes by those who were already diagnosed with diabetes at study entry (prevalent diabetes), and those who were diagnosed during follow-up (incident diabetes).

Preliminary data analysis will employ standard methods, starting with calculation of person years and age-gender standardized cancer incidence rates for all sites and selected sites in non-diabetics, and in prevalent and incident diabetics without prevalent cancer. These methods will also be used to investigate potential confounding variables such as baseline smoking status and body mass index. In our analyses of Specific Aims 2 and 3, we will then proceed to use Cox regression with time-dependent treatment variables. For Specific Aim 2, the model will include current and past levels of Hba1c and blood glucose, investigating the level above selected thresholds cumulated over the past n years, where n may be between 1 and 5. For Specific Aim 3 the model will include current and previous treatments. For example, we will investigate exposure to a specific medication when expressed by the cumulative dose or by the cumulative dose over the past 1–5 years. We will investigate exposure to multiple medications, first by models that postulate independent multiplicative effects on cancer incidence rates (no interaction model); and if there are sufficient data, we will proceed to examine interactions between medications on cancer incidence. Due to data limitations, it is unlikely that anything more than pairwise interactions will be able to be examined satisfactorily. The analyses of glucose-lowering medications will take into account the clinical factors (HbA1c, fasting glucose) that may have triggered the change. We will, for example, use the methods of Walker, White, and Babiker [21] to better evaluate how HbA1c triggers treatment changes. This may or may not be important for cancer incidence analyses, depending on whether HbA1c and fasting plasma glucose are or are not related to cancer incidence (see Specific Aim 2). In analyses of Specific Aims 2 and 3, we will also be cautious of the possibility of reverse causation, whereby early physiological changes before the diagnosis of cancer may cause deterioration in glucose control and subsequent change in diabetes treatment. We will therefore investigate whether early peaks in cancer incidence rates follow a change in treatment, and will examine model results when all cancer diagnoses are moved backwards in time by a set period of 2 years, 1 year, or 6 months.

As outlined above, in specific analyses, multiple independent sensitivity analyses will be used more generally to test our model assumptions and the robustness of our results. We will look at varying time intervals for the piecewise exponential models, stratification for departures from proportional hazards, interactions and time-dependent models including lagged predictors. Propensity scores will be used as an alternative to direct confounder adjustment, to control for confounding by indication, and to check the robustness of conclusions. We will also explore results obtained by applying the recently described “prior event rate ratio” method of Tannen, Weiner, and Xie [22], to the analysis of glucose-lowering medications. Though this method will only be applicable to incident cases of diabetes, its effectiveness in controlling for confounding by indication has been reported.

Cancer prevalent cases will be analyzed separately and will have separate adjustments performed, including separate propensity scoring. This is to overcome potential confounding factors associated with cancer treatments and their residual effects. Furthermore, the risk of second primary neoplasms has not been analyzed in diabetes patients, and might have a different pattern of associations with the disease itself and with the use of glucose-lowering therapies than does the risk of first primaries.

The primary analysis will be performed using PROC PHREG of SAS version 9.2, which allows both Cox-proportional hazards models with time-dependent variables and piecewise exponential models. The piecewise exponential model divides time-at-risk into a set of pre-specified intervals with constant baseline hazard in each interval; the use of multiple intervals allows the hazard to vary with time. Clinically important time dependent covariates for the regression models include age, cholesterol (total, LDL, and HDL), concomitant medications (such as statins, beta-blockers, angiotensin converting enzyme inhibitors, and hormone replacement therapy), smoking, body mass index, blood pressure, co-morbidities (such as cardiovascular disease), and reproductive history. For each continuous variable, we will investigate appropriate transformations and interactions that might improve goodness-of-fit. The proportional hazard assumption will be evaluated using log-log survival plots, examination of Schoenfeld residuals, and testing of interactions between variables and time.

While we can not describe, or even anticipate, every contingency for this comprehensive project, we are confident that the very well qualified team has the expertise to evaluate the data.

This study was approved by the ethics committees of Sheba Medical Center, Tel Hashomer and Clalit Health Services, Israel.

## Discussion

Though randomized controlled trials (RCTs) are considered the gold standard for medical research, large long-term RCTs on the relationship between diabetes and cancer incidence are not feasible (due to high costs and resources), may not be the best way to achieve relevant results (due to the time required, low number of events, and sample selection bias), and are subject to ethical problems (by randomly allocating to treatment groups). Specifically, now that the American College of Physicians (ACP) (February 2012) [23] has strongly recommended monotherapy with metformin as first-line oral therapy for type 2 diabetes, the conduct of RCTs comparing monotherapy glucose-lowering medications, ie. comparing metformin with other oral therapies, would seem unethical.

The advantages of studies based on health insurance administrative databases have become recognized over recent years [24,25]. Moreover, historical cohort studies afford timely and ethical means of comparing effects of glucose-lowering medications, albeit with methodological challenges. Recently, a nested case control [26] and cross-sectional study [7] adjusted for a number of confounding variables before concluding that metformin has a protective effect on cancer outcomes. However, those investigations did not compare the diabetes patients to a non-diabetic population, nor analyze by cancer type. Currie et al., on the other hand, recently reported decreased mortality among cancer patients using metformin, even compared to those without diabetes, and increased survival among metformin users for most cancer sites, but not for breast cancer [27]. The fact that increased breast cancer was also reported among insulin users, compared to other cancer types [19,28], suggests that such differences may be due to confounding factors rather than to the effects of medications. Most studies did not account for surveillance bias, ie. the possibility that the detection of cancer may differ between those with and without diabetes, resulting in a spurious difference in cancer incidence between the two groups. The observation that women with diabetes were found to screen less for cancer than women without [29,30] highlights the importance of mitigating surveillance bias.

The current study will undertake various means to deal with the challenges posed by recent investigations, including comparison to a large population of individuals without diabetes in a nationwide population, and accessing a large number of potentially confounding factors and data on cancer screening.

Statistical power is expected for a number of monotherapies and combination treatments among individuals newly diagnosed with diabetes, thereby controlling for the duration of diabetes and for all glucose-lowering medications used during the course of their treatment. Temporal relationships between diabetes and cancer will be considered. A number of aspects of the study design are expected to elucidate the complex relationship between diabetes and cancer, and possible effects of glucose-lowering medications: analysis of data preceding diabetes incidence, the inclusion of “no glucose-lowering medications” as an exposure, and the inclusion of all individuals with and without diabetes who were insured by one very large health maintenance organization during a ten year period.

## Competing interests

The authors declare that they have no competing interests.

## Authors’ contributions

RD conceived of the study, participated in its design and drafted the manuscript. RB is in charge of acquisition of data, participated in the design of the study and helped to draft the manuscript. PB participated in the design of the study and helped to draft the manuscript. LKB participated in the design of the study and helped to draft the manuscript. SW participated in the design of the study and will perform the statistical analysis. LF participated in the design of the study and the design of the statistical analysis plan, and helped to draft the manuscript. MG contributed to acquisition of data and contributed to draft the manuscript. JR participated in the design of the study and helped to draft the manuscript. RT participated in the design of the study and helped to draft the manuscript. DL participated in the design of the study and helped to draft the manuscript. All authors read and approved the final manuscript.

## Pre-publication history

The pre-publication history for this paper can be accessed here:

http://www.biomedcentral.com/1471-2407/12/364/prepub
